# Progress of Medical Science in Germany

**Published:** 1875-02-01

**Authors:** Edmund J. Doering


### Translations.

### PROGRESS OF MEDICAL SCIENCE IN GERMANY.

By Edmund J. Doering, M.D.

I. Snow as a Cleansing and Styptic Application for Surgical Wounds, by Dr. Hasse (*Centralblatt fur Chirurgie*, No. 38). II. Pleuritis; Empyema; Paracentesis Thoracis; Cure, by Dr. Blaschko (*Allg. Med. Central Zeitung*, No. 100). III. Medical Examinations in Germany.

I.

SINCE we are able to perform operations on the extremities without the loss of a drop of blood by Prof. Esmarch's method, we experience much more than formerly the great inconvenience of hæmorrhage whenever we have to operate on parts of the body where a compression of the blood-vessels is inadmissible; and still more so, if at the same time the capillaries and veins of the parts are overloaded and distended. Especially is this true of tracheotomy, for often it is essential here to hasten the various steps of the operation, rendered difficult by troublesome hæmorrhage.

Several times we had to perform this operation on winter days, when there had been a recent fall of snow. We gathered some of the latter, and used it instead of sponges, for the cleansing of the wound. When a handful of soft snow is pressed firmly for a few seconds on the wound, it will present a true cast thereof on being removed, leaving the cut surface free of blood, and benumbed for a while. The blood is absorbed much better by snow than by a sponge —and furthermore, the uniform compression, and the local influence of cold, causes the snow to act as an astringent on the cut capillaries and smaller vessels—and finally, through this latter property, also, as an anæsthetic on the nerves.

Thus, by repeated application of soft snow, we have a substitute for Prof. Esmarch's method, allowing a rapid and safe termination of the operation, and enabling the surgeon to operate with less chloroform than is usually required.

It is to be regretted that we cannot always have access to this excellent resource; for even at the proper season of the year, only quite soft snow is fit for our purpose. The snow must also be gathered very carefully, which is best done by lifting it up from the ground with a light shovel, and throwing it loosely on a flat dish.

II.

Since the introduction of improved instruments for the tapping of pleuritic effusions, the number of recoveries from pyothorax and hydrothorax have largely increased, especially of the latter disease, where in operating, it is of great consequence to prevent the entrance of air, which can be done very effectually by means of the aspirator. Thoracentesis must, therefore, always be preferred to a tardy absorption or a spontaneous evacuation of the effusion.

To insure a recovery, many operators have been compelled not only to perform thoracentesis repeatedly on the same patient, but also to wash out the pleural cavity by means of injections. For this reason, the following case of empyema may be of interest, showing a complete recovery within a short time, after a single operation for the removal of the pus, with careful after-treatment.

Miss B., aged fifteen; has had fair health until her present illness, which commenced suddenly after exposure to cold, in June, 1874, with all the symptoms of an acute attack of pleurisy. The attending physician did not succeed in preventing an abundant effusion from taking place, or in promoting the absorption of the same during a period of two months. We were called to see the patient in the middle of August. We found her looking pale and greatly emaciated. Pulse quick; temperature increased; night-sweats, and an abundant catarrhal expectoration. The patient complained of tightness across the chest, sleeplessness, and a feeling of anxiety. Appetite lost; menstruation arrested. Physical examination showed the left side of the chest enlarged, bulging of the intercostal spaces, with complete dullness on percussion from the second rib downwards. Respiratory sounds absent: heart transposed to the right. On the left side of the chest, portions of the fourth and fifth rib projected irregularly, justifying apprehensions of caries and external discharge of the fluid contents of the thoracic cavity.

The fever and cough continuing, the emaciation progressing, the weakness increasing, with a copious discharge of sputa, and the inability to cause the absorption of the purulent fluid, either by external or internal remedies, thoracentesis had to be thought of, as offering the only chance of saving the patient's life. Prof. Waldenburg was called in consultation, and fully agreed with us in the advisability of the operation. We performed the operation without the administration of chloroform to the patient. The tap trocar was passed into the pleural cavity between the fifth and sixth rib, brought in connection with the aspirator, and about twenty-five ounces of a creamy pus drawn off. The wound was closed by a piece of sticking plaster. No injections of any fluids were made into the pleural cavity, and the opening was completely closed on the following day, no more pus having been discharged.

The patient felt immediately relieved after the operation; she coughed less, and rested well the following night. Quinine, iron, and beef-tea, were prescribed, and the patient sent to the country.

Prof. Waldenburg, finding on physical examination, still a portion of the purulent fluid within the pleural cavity, feared that the operation would have to be repeated. But this did not prove to be necessary, for within four weeks, no trace of any fluid could be detected, the patient having completely recovered. The rapidity of the cure was certainly greatly accelerated by the residence in the country, the salubrious country air, leaving the patient mostly out doors, the internal use of cod-liver oil, and the external application of tincture of iodine.

At present, the young lady is in the enjoyment of perfect health, having a good appetite, getting stronger, and being free of cough. In this connection, we desire to call attention to the following points:

(1.) The differential diagnosis between pyo and hydrothorax, must be based more on the general symptoms, than on physical examination alone. If the fever does not subside after the use of quinine and digitalis, and emaciation progresses, night-sweats set in, then the presence of pus in the pleural cavity is established beyond a doubt.

(2.) Chloroform must not be administered, as the patient, by repeated coughing, can materially facilitate the discharge of the pus.

(3.) Injections of warm water, or of any other fluid, are not necessary.

(4.) A portion of the pus may remain in the pleural cavity, without necessarily retarding a complete recovery.

(5.) Thoracentesis is the best method of saving life in such desperate cases.

III.

The graduates in medicine of the nine Universities of Prussia, Germany, are compelled by law, to present themselves before a "State Board of Medical Examiners," for examination, before they can be licensed to practice medicine in that state. This same law also exists, and is rigidly enforced in the other states of the German Empire; likewise in Austria, France, England, and in nearly all of the other prominent countries of the world, with the exception of the United States of America.

The following table shows the result of the examinations in Prussia during the past year, and conveys also an idea, how rigid these examinations are, for about twenty-five per cent of the candidates were rejected; and we might further add, that no candidate is allowed to go up for examination, unless he can prove, by certificates, that he has attended at least eight courses of medical lectures — equivalent to four years study:

1873–74.

| Universities. | No. of Candidates. | Passed. | Rejected. |
|---|---|---|---|
| Berlin, | 124 | 89 | 35 |
| Bonn, | 39 | 33 | 6 |
| Breslau, | 37 | 32 | 5 |
| Goettingen, | 34 | 32 | 2 |
| Greifswald, | 81 | 61 | 20 |
| Halle, | 63 | 49 | 14 |
| Kiel, | 21 | 18 | 3 |
| Koenigsburg, | 45 | 25 | 20 |
| Marburg, | 33 | 30 | 3 |
| Total, | 477 | 369 | 108 |

The sum total of physicians licensed in the whole German Empire for the year 1874, is only 660.

During the same year, the innumerable medical colleges of the United States of America, graduated three thousand students.

In conclusion, we add for comparison, the following table:

1874.

| Country. | No. of Inhabitants, | Practitioners Licensed in 1874. |
|---|---|---|
| Germany, | 42,000,000 | 660 |
| United States, | 40,000,000 | 3,000 |

Further comment is unnecessary.

